# Higher Number of Tumor-Infiltrating PD-L1+ Cells Is Related to Better Response to Multikinase Inhibitors in Hepatocellular Carcinoma

**DOI:** 10.3390/diagnostics13081453

**Published:** 2023-04-18

**Authors:** Ji Won Han, Ji Hoon Kim, Dong Hyun Kim, Jeong Won Jang, Si Hyun Bae, Jong Young Choi, Seung Kew Yoon, Jaegyoon Ahn, Hyun Yang, Pil Soo Sung

**Affiliations:** 1 The Catholic University Liver Research Center, College of Medicine, The Catholic University of Korea, Seoul 06591, Republic of Korea; tmznjf@catholic.ac.kr (J.W.H.); jihoon23@catholic.ac.kr (J.H.K.); moocimay@naver.com (D.H.K.); garden@catholic.ac.kr (J.W.J.); baesh@catholic.ac.kr (S.H.B.); jychoi@catholic.ac.kr (J.Y.C.); yoonsk@catholic.ac.kr (S.K.Y.); 2 Division of Gastroenterology and Hepatology, Department of Internal Medicine, College of Medicine, Seoul St. Mary’s Hospital, The Catholic University of Korea, Seoul 06591, Republic of Korea; 3 Division of Gastroenterology and Hepatology, Department of Internal Medicine, College of Medicine, Eunpyeong St. Mary’s Hospital, The Catholic University of Korea, Seoul 03382, Republic of Korea; 4 Department of Computer Science & Engineering, Incheon National University, Incheon 22573, Republic of Korea; jgahn@inu.ac.kr

**Keywords:** hepatocellular carcinoma, sorafenib, lenvatinib, CD3, PD-L1, CD68

## Abstract

Multikinase inhibitors (MKIs) such as sorafenib and lenvatinib are first-line treatments for unresectable hepatocellular carcinoma (HCC) and are known to have immunomodulatory effects. However, predictive biomarkers of MKI treatment in HCC patients need to be elucidated. In the present study, thirty consecutive HCC patients receiving lenvatinib (*n* = 22) and sorafenib (*n* = 8) who underwent core-needle biopsy before treatment were enrolled. The associations of CD3, CD68, and programmed cell death-ligand-1 (PD-L1) immunohistochemistry with patient outcomes, including overall survival (OS), progression-free survival (PFS), and objective response rate (ORR), were evaluated. High and low subgroups were determined according to median CD3, CD68, and PD-L1 values. Median CD3 and CD68 counts were 51.0 and 46.0 per 20,000 µm^2^, respectively. The median combined positivity score (CPS) of PD-L1 was 2.0. Median OS and PFS were 17.6 and 4.4 months, respectively. ORRs of the total, lenvatinib, and sorafenib groups were 33.3% (10/30), 12.5% (1/8), and 40.9% (9/22), respectively. The high CD68+ group had significantly better PFS than the low CD68+ group. The high PD-L1 group had better PFS than the low subgroup. When we analyzed the lenvatinib subgroup, PFS was also significantly better in the high CD68+ and PD-L1 groups. These findings suggest that high numbers of PD-L1-expressing cells within tumor tissue prior to MKI treatment can serve as a biomarker to predict favorable PFS in HCC patients.

## 1. Introduction

Hepatocellular carcinoma (HCC) accounts for approximately 80% of all primary liver cancers and is one of the most common causes of cancer-related mortality [1]. The first-line treatment for unresectable HCC is a combination treatment of atezolizumab and bevacizumab (AB). Before the AB combination therapy became the first-line treatment, multikinase inhibitors (MKIs) such as sorafenib or lenvatinib were used as first-line treatments for unresectable, advanced HCC [2]. In cases not suited to AB, such as transplant recipients, those with autoimmune diseases, or those at high risk of variceal bleeding, sorafenib or lenvatinib is still considered the treatment of choice [3].

Interestingly, a recent propensity-matched (PSM) study that analyzed patients with non-viral HCC reported that lenvatinib resulted in better overall survival (OS) and progression-free survival (PFS) than AB treatment [4]. A recent large-scale, global, real-world study also reported that lenvatinib might be better than AB in advanced HCC in terms of objective response rate (ORR) [5]. Another real-world study using PSM and inverse probability of treatment weighting reported that two first-line treatments were comparable in terms of OS and PFS in unresectable HCC [6]. These findings suggest that MKIs might still have a role in treating unresectable HCCs as a first-line treatment. Further comparative studies are needed to validate these results. The prediction of clinical outcomes using various biomarkers in ICIs and MKIs, which might also be helpful in choosing the proper regimen among ICIs and MKIs, also requires further study.

Both ICIs and MKIs have immunomodulatory effects [7]. Sorafenib can improve antitumor immune responses by regulating tumor-associated macrophages (TAMs) [8], or enhancing T-cell responses [9]. Lenvatinib also has immunomodulatory effects, which were found to decrease monocytes and macrophages but augment T-cell responses in an in vivo mouse model [10]. Lenvatinib also targets fibroblast growth factor receptor (FGFR), resulting in enhanced anti-programmed cell death 1 (PD-1) therapy [11]. A small human study comprehensively analyzed the dynamic change of immune cells and cytokines in the peripheral blood after lenvatinib treatment in HCC [12]. Treatment resulted in a decrease in the frequency of T-helper and T-regulatory cells, but there was a significant increase in cytotoxic T lymphocytes. The cytokine profiles showed an increase in interleukin-2 (IL-2), IL-5, and IFN-γ, while there was a decrease in other cytokines, such as IL-6, IL-10, tumor necrosis factor-α (TNF-α), and transforming growth factor (TGF-β). Furthermore, the expression of PD-1 and TIM-3 on cytotoxic T lymphocytes significantly decreased, and the expression of TIM-3 and CTLA-4 also decreased on T-regulatory cells. The low CTL/Treg ratio was found to be associated with a poor outcome in HCC patients.

Previous studies characterized HCC subtypes as “immunocompetent”, “immunosuppressive”, and “immunodeficient” [13,14]. The first subtype is characterized by a robust infiltration of T cells (CTLs and Th1 cells) and TAMs, with an M1-dominant phenotype. CD68 is a glycoprotein predominantly expressed in macrophages, which aids in recognizing targets by attaching to specific lectins or selectins in tissues or organs. As a universal macrophage marker, CD68 allows for the detection of all macrophage types, irrespective of their phenotype. M1 macrophages exhibit anticancer and proinflammatory properties, whereas M2 macrophages are linked to cancer-promoting and immune-suppressing effects [15]. TAMs express PD-L1, which reflects the immunogenic nature of the tumor. This phenotype is expected to respond well to ICI therapies. In the second subtype, immunosuppressive cells, including TAMs, are highly infiltrated, while T-cell infiltration is low. TAMs may express PD-L1 but at a lower level than that seen in cells with an immunocompetent phenotype. This phenotype may not respond well to immune checkpoint inhibitor therapies and may require combination treatments. The third subtype is referred to as the “immunodeficient” subtype. The infiltration of T cells and TAMs is poor, possibly due to poor tumor immunogenicity. This subtype may not respond to immune-based therapy unless antigen release by the locoregional or systemic therapies results in local inflammation sufficient to cause immune cell infiltration. Furthermore, whether these different subtypes influence the response of MKIs is unknown.

Therefore, analyzing immune cell population for HCC patients receiving MKIs might be helpful for predicting clinical outcome. We reported that T cells and PD-L1-expressing macrophages can predict lenvatinib in HCC patients, although the sample size was small [16]. Previous studies have suggested that clinical, serum, and tissue markers, including the VEGF receptor or c-met, can predict responses to sorafenib (reviewed in [17]), while tumor FGFR4 expression can predict responses to lenvatinib [18]. However, whether the immune-cell population can be a biomarker for MKIs still needs to be studied. Therefore, in the present study, we evaluated the impact of CD3+, CD68+, and PD-L1 expression on MKI responses and patient outcomes, investigating tissue samples from patients with unresectable HCC via immunohistochemistry.

## 2. Materials and Methods

### 2.1. Study Design and Population

In this study, we retrospectively reviewed the medical records of 30 patients with unresectable hepatocellular carcinoma (HCC) who underwent core-needle tumor biopsy between December 2017 and June 2022 at Seoul St. Mary’s Hospital and Eunpyeong St Mary’s Hospital. Of the 30 patients, 8 were treated with sorafenib, and 22 were treated with lenvatinib. The diagnosis of HCC was made based on histological and/or radiological findings, which included imaging studies such as multiphasic computed tomography and magnetic resonance imaging [3]. Imaging studies including multiphasic computed tomography and magnetic resonance imaging were included in the diagnosis of HCC. This study was approved by the Institutional Review Boards of Seoul St. Mary’s Hospital and Eunpyeong St. Mary’s Hospital (XC21RIDI0138). The study conformed to the ethical guidelines of the Helsinki Declaration. Written informed consent was obtained from each patient prior to enrollment. Patients with a viral etiology were defined as those with either hepatitis B virus or hepatitis C virus infections.

### 2.2. Immunohistochemistry

Immunohistochemistry obtained by core-needle liver biopsy samples was used in this study. Immunohistochemistry was performed as previously described [19]. In detail, A 5-micrometer-thick cross section of a paraffin-embedded block was transferred onto a silanized glass slide, then deparaffinized with xylene and rehydrated using a graded series of alcohols. To retrieve antigens, the sample was heated in 0.01 M citrate buffer (pH 6.0) for 20 min using a microwave vacuum histoprocessor (RHS-1; Milestone, Bergamo, Italy) until it reached a final temperature of 121 °C. To prevent endogenous peroxide activity, the section was incubated with hydrogen peroxide (3%) in methanol for 10 min. Next, the slides were incubated with antibodies against CD3 (Abcam), CD68 (clone: KP1, Dako, Carpinteria, CA, USA), and PD-L1 (clone: 22C3, Dako). After washing, the EnVision+ system HRP-labelled polymer (Dako) was applied to the slides at 24 °C for 5 min. The slides were then treated with 3,3′-diaminobenzidine for 5 min and counterstained with hematoxylin.

We then counted the CD3- or CD68-positive cell number per 20,000 µm in HCC tissue samples, and a combined positive score (CPS) was calculated for the PD-L1 expression, as previously described [20]. We designated patients with higher or equal to median cell counts as the “high” cell-count group and patients with lower than median cell counts as the “low” group.

### 2.3. Response Evaluation

The treatment response was evaluated every 2 to 3 months after treatment according to the modified Response Evaluation Criteria in Solid Tumors (mRECIST) criteria [21,22]. Tumors with no arterial enhancement were categorized as tumors with complete response. Tumors in which the sum of the diameters of viable lesions was reduced by > 30% were defined as tumors with partial response (PR). Cases in which the sum of the viable lesions increased by >20% were classified as progressive disease (PD) cases. Cases that did not meet the criteria for partial response or progressive disease were defined as stable disease (SD) cases.

### 2.4. Statistical Analyses

SPSS version 26 software (IBM Corp., Armonk, NY, USA) was used for data analyses. The categorical variables associated with the two groups were compared using chi-square tests, and the continuous variables were assessed using an independent *t*-test. The Kaplan–Meier method was used for the survival analyses, and survival curves were compared using log-rank tests. Factors associated with survival were analyzed using Cox proportional hazards regression. Correlation between two parameters was analyzed by Spearman test. Statistical significance was set at *p* < 0.05.

## 3. Results

### 3.1. Patient Characteristics and Outcomes

This study enrolled a total of 30 patients; their baseline clinical characteristics are presented in Table 1. The most prevalent etiology of HCC was hepatitis B virus infection (60%), followed by alcohol (20%), hepatitis C infection (3.3%), and autoimmune hepatitis (16.7%). The median serum alpha-fetoprotein (AFP) level was found to be 160 ng/mL. The mean largest intrahepatic tumor size was 9.4 cm, with 21 patients (70%) exhibiting multiple tumor lesions and the remaining 9 (30%) with a single tumor lesion. Of the total patient population, 20 patients (66.7%) did not exhibit portal vein invasion, whereas 10 patients (33%) did. In addition, 14 patients (46.7%) had extrahepatic metastasis. Nine patients (30.0%) showed signs of clinically significant portal hypertension including varices or ascites. In terms of Child–Pugh scores, 17 patients (56.7%), 7 patients (23.3%), and 5 patients (16.7%) had scores of 5, 6, and 7, respectively, while only 1 patient (3.3%) had a Child–Pugh score of 8. Of the 30 patients, 23 (76.7%) had a history of previous treatments, including surgical treatment and local therapy such as transarterial chemoembolization. In accordance with the Barcelona Clinic Liver Cancer staging system, 12 patients (40%) were classified as Stage B, while 18 patients (60%) were classified as Stage C. Median CD3 and CD68 counts were found to be 51.0 and 46.0 per 20,000 µm^2^, respectively. Additionally, the median CPS of PD-L1 was found to be 2.0. We observed that the median overall survival (OS) and median progression-free survival (PFS) were 17.6 months and 4.4 months, respectively. When we compared baseline characteristics between the low and high PD-L1 groups (Table 2), only CD68 counts displayed a significant difference, with higher counts in the high PD-L1 subgroup (*p* = 0.001).

### 3.2. Factors Associated with Patient Outcomes

In order to identify factors associated with OS and PFS, we conducted a Cox regression analysis. Our analysis revealed that with respect to OS, a Child–Pugh score of greater than 5 was the sole factor significantly associated with poor OS, both in the univariate analysis (hazard ratio (HR)= 4.17, *p* = 0.03) and multivariate analysis (HR = 4.27, *p* = 0.02) (Table 3).

Subsequently, we conducted a Cox regression analysis to determine the factors associated with PFS, as presented in Table 4. Our results demonstrate that high CD68+ cell counts were significantly associated with improved PFS, with an HR of 0.26 (*p* = 0.02). Additionally, high PD-L1+ CPS was found to be significantly associated with better PFS, with an HR of 0.33 (*p* = 0.03). Conversely, patients with a Child–Pugh score of greater than 5 were significantly associated with poorer PFS, with an HR of 3.071 (*p* = 0.04). However, in the multivariate analysis, no significant factor was found to be associated with PFS.

### 3.3. Difference in PFS according to the Expression of Immunologic Markers

**Figure 1:**
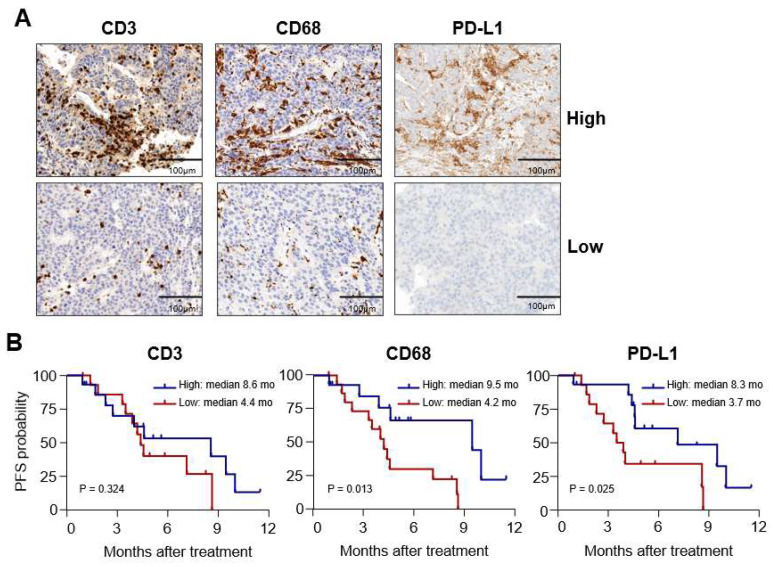
Differences in progression-free survival (PFS) according to the expression of immunologic markers. (**A**) Representative immunohistochemistry findings for CD3, CD68, and programmed cell death-ligand-1 (PD-L1) in hepatocellular carcinoma (HCC) biopsy samples. (**B**) Kaplan–Meier curves showing PFS in high and low subgroups determined by median cell counts of each marker (as shown).

To further investigate the association between the expression levels of CD3, CD68, and PD-L1 within tumor *t*issues and patient outcomes, we divided the patient population into subgroups based on the median values of respective markers, as shown in Table 1. Representative immunohistochemistry findings are presented in Figure 1A. We found that there was no significant difference in OS between subgroups for each marker (data not shown). However, when we compared PFS, we observed that the high CD3+ subgroup did not have a significantly different PFS period compared to the low CD3+ subgroup (*p* = 0.32; Figure 1B, left). In contrast, the high CD68+ subgroup had significantly better PFS than the low CD68+ subgroup (median 9.5 months versus 4.2 months, *p* = 0.01; Figure 1B, middle). Similarly, the high PD-L1 subgroup also demonstrated a better PFS period than the low PD-L1 subgroup (median 8.3 months versus 3.7 months, *p* = 0.02; Figure 1B right).

### 3.4. Difference in the Expression of Immunologic Markers according to Treatment Response

**Figure 2:**
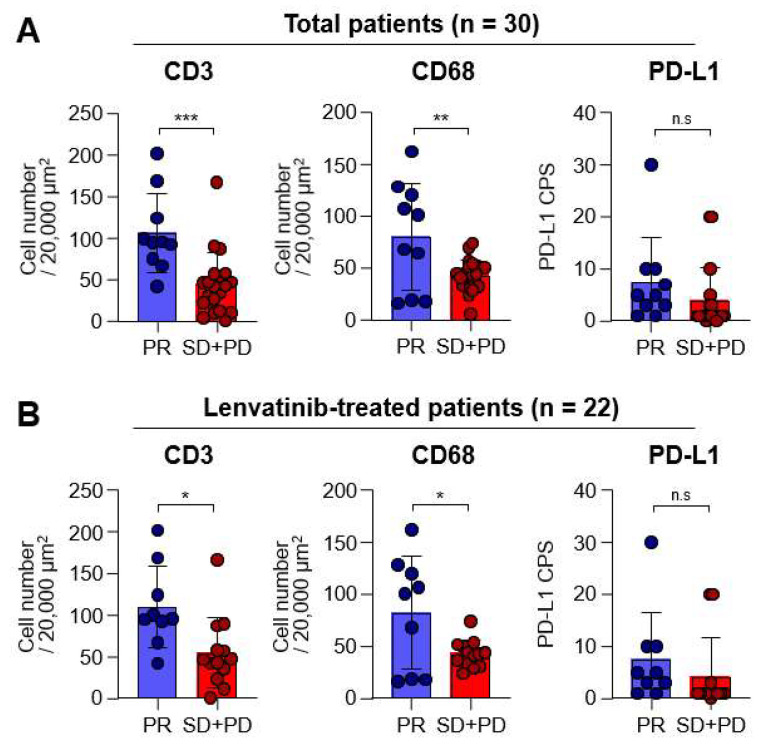
Differences in the expression of each marker according to the best responses. Graph showing the objective response rates of total (*n* = 30) and lenvatinib (*n* = 22) groups. (**A**) Graphs showing the CD3+ and CD68+ cell numbers, as well as the programmed cell death-ligand-1 (PD-L1) combined positive score (CPS) in total patients with partial response (PR; *n* = 10) and stable disease (SD) plus progressive diseases (PD; *n* = 20) in the total group. (**B**) Graphs showing the CD3+ and CD68+ cell numbers, as well as PD-L1 CPS, in lenvatinib-treated patients with PR (*n* = 9) and SD plus PD (*n* = 13). n.s., not significant; * *p* < 0.05; ** *p* < 0.01; *** *p* < 0.001.

In order to explore whether the expression of each marker differed according to the best responses following MKI treatments, we conducted further analysis. ORRs for the total patient population and the sorafenib and lenvatinib groups, were 33.3% (10/30), 12.5% (1/8), and 40.9% (9/22), respectively, and no statistically significant differences were found between the sorafenib and lenvatinib groups (*p* = 0.14). Upon dividing patients into PR and SD plus PD groups, we found that cell counts for CD3 and CD68 in the PR group were significantly higher than those in the SD + PD group (*p* < 0.001 and *p* = 0.005, respectively; Figure 2A, left and middle, respectively). However, no significant differences were observed in PD-L1 CPS expression between the two groups (Figure 2A, right). Moreover, a similar trend of higher CD3 and CD68 counts in the PR group was also observed in the lenvatinib subgroup (*p* = 0.011 and *p* = 0.020, respectively; Figure 2B).

### 3.5. Correlation between CD3, CD68, PD-L1, and Clinical Parameters

**Figure 3:**
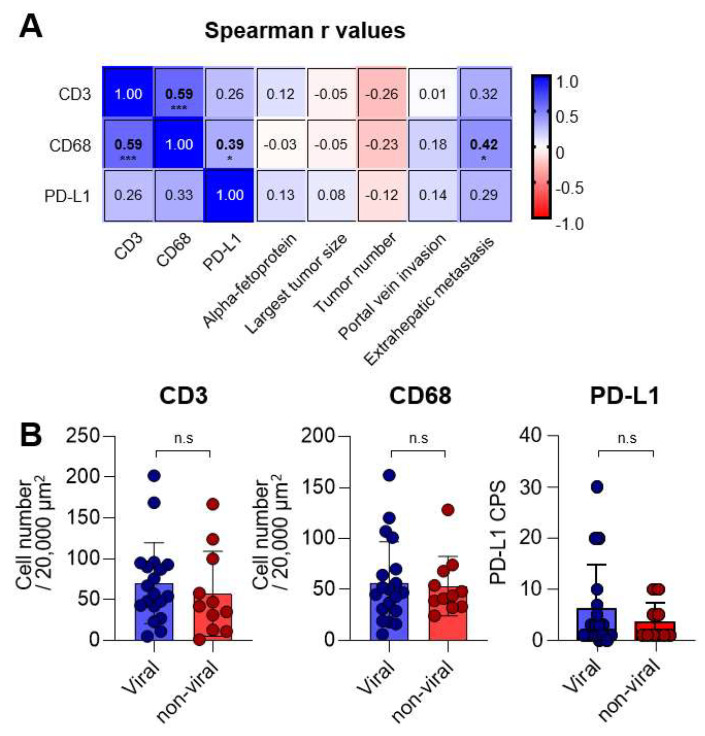
Correlations between CD3, CD68, programmed cell death-ligand-1 (PD-L1), and clinical parameters. (**A**) Matrix presenting the Spearman r values between each immunologic and clinical parameter. (**B**) Graphs showing the CD3+ and CD68+ cell numbers, as well as PD-L1 CPS, in MKI-treated patients with viral (n = 19) and non-viral (n = 11) etiologies. n.s., not significant; * *p* < 0.05; *** *p* < 0.001.

To further investigate the relationship between CD3, CD68, PD-L1, and clinical parameters, we conducted a correlation analysis (as presented in Figure 3A). Our analysis revealed a positive correlation between intratumoral CD3 and CD68 expressions (r = 0.59, *p* < 0.001), suggesting that T-cell and macrophage infiltration within HCC tissues might be significantly associated.

Furthermore, we observed a positive correlation between PD-L1 CPS and CD68 expression (r = 0.39, *p* = 0.01), indicating that CD68+ macrophages might be an important PD-L1-expressing population. It is worth noting that more intratumoral CD68 expression was found to be associated with the presence of extrahepatic metastasis (r = 0.42, *p* = 0.02). These findings suggest that CD3, CD68, and PD-L1 expressions might not be significantly affected by tumor-related factors, except for the presence of extrahepatic metastasis. In addition, we analyzed the expressions of CD3, CD68, and PD-L1 based on viral and non-viral etiologies (Figure 3B). As a result, no difference was observed between the viral (n = 19) and non-viral subgroups (n = 11) in the expression of CD3, CD68, and PD-L1, suggesting that these markers may not be influenced by the etiologies of HCC.

## 4. Discussion

In patients with HCC, AB combination treatment is considered the first-line systemic treatment in the absence of contraindications [23]. In cases in which this treatment is not feasible, MKIs such as sorafenib or lenvatinib are considered. Therefore, it is crucial to identify patients who can achieve favorable outcomes following MKI treatment, although it is currently unclear how this can be achieved. In this study, we hypothesized that the levels of T cells, TAMs, and PD-L1 within biopsy tissues prior to MKI treatment, as determined by simple immunohistochemistry, can serve as biomarkers to predict patient outcomes. Our study demonstrates that these tissue-based immunologic markers can be used as predictive biomarkers in HCC patients treated with sorafenib or lenvatinib. In particular, high numbers of CD68- and PD-L1-expressing cells within tumors were found to be associated with better progression-free survival (PFS), while higher numbers of CD3 and CD68 were found to be related to objective response. These findings warrant validation in larger studies in the future. Identifying predictive biomarkers could facilitate the development of more personalized treatment strategies, potentially leading to better outcomes for patients with HCC.

HCC development is associated with a progressive dysfunction of both the innate and adaptive immune systems. The resulting increase in regulatory components and immune suppressors contribute to the formation of an immunosuppressive tumor microenvironment (TME) [24]. Among these immune suppressors, M2-polarized TAMs are of particular importance in HCC. They are known to contribute to the immunosuppressive TME by producing immunosuppressive cytokines and inhibiting T-cell activity [25]. TAMs are also a significant source of PD-L1 expression in HCC, which further contributes to immune evasion by the tumor [26]. A previous study revealed that lenvatinib exhibited antitumor activity via a reduction of the monocyte and macrophage populations in mice [10]. Similarly, another study using a mouse model reported that lenvatinib treatment decreased the TAM population, resulting in enhanced antitumor immunity when administered followed by anti-PD-1 treatment [27]. Therefore, our results, which show an association between CD68 or PD-L1 expression and lenvatinib treatment, require further examination to fully understand the dynamic changes in the phenotype and number of macrophages following MKI treatment. A recent study revealed that E-twenty-six-specific sequence variant 4 (ETV4) increases PD-L1 expression, leading to enhanced TAM infiltration and suppressed T-cell accumulation, ultimately resulting in HCC metastasis [28]. Importantly, ETV4 expression was upregulated by fibroblast growth factor 19 (FGF19) and fibroblast growth factor receptor 4 (FGFR4). Since the major molecular target of lenvatinib is FGFR, this mechanism might be related to our observations, although it was primarily studied using in vitro cell lines and in vivo mouse models.

**Figure 4:**
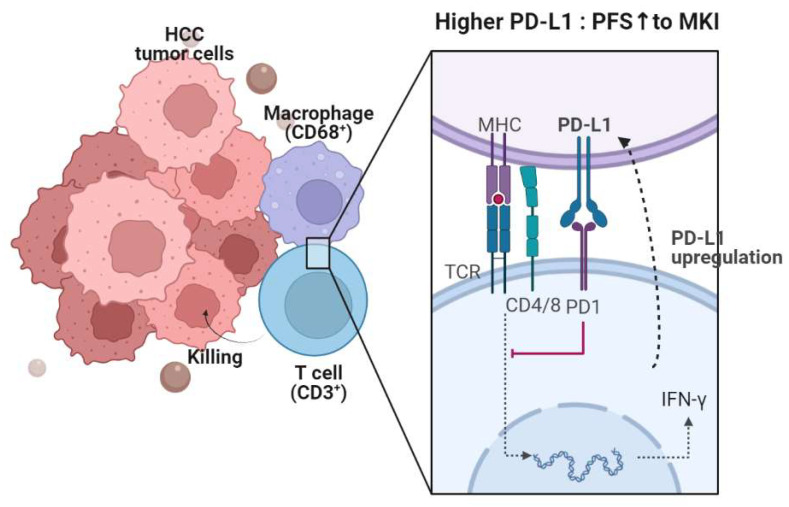
A synopsis of the present research. PD-L1 is expressed in CD68+ macrophages within the HCC microenvironment. The PD-1-PD-L1 axis leads to T-cell exhaustion, consequently reducing antitumor function. However, PD-L1 is upregulated upon T-cell activation and production of IFN-γ and cytotoxic molecules. The expressions of CD68 and PD-L1 are linked to progression-free survival (PFS), while CD3 and CD68 expressions are associated with the objective response rate (ORR).

Exhausted T cells show upregulated expression of several inhibitory receptors, including PD-1, and its ligand PD-L1 is expressed on various cells, including tumor cells and antigen-presenting cells [29]. Previous studies have reported immunomodulatory activity of lenvatinib in terms of adaptive immunity [10]. A recent study found that lenvatinib treatment reduced regulatory T cells and helper T cells, whereas cytotoxic T cells were increased [12]. Another study reported that renal cell carcinoma inoculated into mice was more responsive to lenvatinib when the tumor microenvironment consisted of T cells [30]. On the other hand, sorafenib combined with HER-2-targeted vaccination increased T-cell immunity in a breast cancer in vivo model [9]. These findings suggest that T cells might be closely related to the effect of MKIs, either directly or indirectly. In the present study, we observed that a high intratumoral CD3+ cell count is correlated with favorable responses following MKI treatment, which is consistent with previous studies. Furthermore, a high PD-L1 combined positive score (CPS) was found to be associated with better progression-free survival (PFS). These findings suggest that pre-existing T cells that were dysfunctional before MKI treatment may be restored. Additionally, the number of CD3+ cells in the tumor microenvironment was found to be correlated with the number of CD68+ cells, which raises the further necessity of evaluating macrophage populations. Figure 4 shows a schematic representation of the effects of PD-1 signaling on T cells, in conjunction with PD-L1 and macrophages, as well as their prognostic role in MKI treatment.

The study results reported herein present some limitations that should be taken into consideration. One of the limitations is the small number of cases examined, which may have affected the statistical power of the findings. This might be attributable to the study’s retrospective design and the constraints associated with liver biopsy. Future larger, prospective studies are warranted. Furthermore, the lack of mechanistic studies made it difficult to fully understand the underlying biological mechanisms that contribute to the observed changes in immune cell populations and cytokine profiles. Therefore, further studies are needed to elucidate the underlying mechanisms involved in the observed changes. Additionally, it should be noted that the study population mostly consisted of individuals with chronic hepatitis B; therefore, the results may not be generalizable to non-viral HCC cases, making it essential to validate these findings in larger and more diverse populations, including those with non-viral HCC. Further studies with larger sample sizes and more diverse populations are required to confirm and extend these findings and to better understand the role of immune cell populations and cytokines in the pathogenesis of HCC. We were also unable to analyze the subpopulations of CD3+ and CD68+ cells, which consist of both antitumor and immunosuppressive subsets. A more comprehensive phenotypic analysis addressing this issue could provide insight into their roles in predicting prognosis and antitumor effects in HCC patients receiving MKIs.

In conclusion, we report that high numbers of CD68- and PD-L1-expressing cells within tumor tissue prior to MKI treatment can be a biomarker used to predict PFS in patients with HCC. Our results highlight the importance of immunologic examinations prior to sorafenib or lenvatinib treatment. Future larger, prospective studies are needed.

## Figures and Tables

**Table 1:** Baseline characteristics.

	Total (*n* = 30)
Age	64.3 ± 13.3
Male gender	26 (86.7)
Treatment	
Sorafenib	8 (26.7)
Lenvatinib	22 (72.3)
Etiology	
Hepatitis B virus	18 (60.0)
Hepatitis C virus	1 (3.3)
Alcohol	6 (20.0)
Autoimmune hepatitis	5 (16.7)
Alpha-fetoprotein, ng/mL	160 (32.5, 9441.0)
Largest intrahepatic tumor size (cm)	9.4 ± 6.2
Intrahepatic tumor number	
Single	9 (30.0)
Multiple	21 (70.0)
Portal vein invasion	10 (33.3)
Extrahepatic metastasis	14 (46.7)
Asparatae aminotransferase (U/L)	60.2 ± 48.1
Alanine aminotransferase (U/L)	33.6 ± 17.5
Total bilirubin (mg/dL)	1.1 ± 0.7
Albumin (g/dL)	3.8 ± 0.6
Platelet count (10^9^/L)	167.6 ± 74.4
INR	1.1 ± 0.2
Creatinine (mg/dL)	0.9 ± 0.5
Clinically significant portal hypertension	9 (30.0%)
Child–Pugh score	
5	17 (56.7)
6	7 (23.3)
7	5 (16.7)
8	1 (3.3)
Previous treatment history	23 (76.7)
Barcelona Clinic Liver Cancer stage	
B	12 (40.0)
C	18 (60.0)
CD3 (Cell number/20,000 µm^2^)	51.0 (30.0, 93.5)
CD68 (Cell number/20,000 µm^2^)	46.0 (31.8, 68.5)
PD-L1 (Combined positive score)	2.0 (1.0, 6.5)
Median overall survival, days	527
Median progression-free survival, days	132

Data are presented as *n* (%), mean ± SD, or median (quartiles). PD-L1, programmed cell death-ligand-1.

**Table 2:** Comparison of baseline characteristics between the high and low PD-L1 subgroups.

	High (*n* = 15)	Low (*n* = 15)	*p-*Value
Age	62.8 ± 12.6	65.7 ± 14.2	0.555
Male gender	15 (100.0)	11 (73.3)	0.107
Treatment			1.000
Sorafenib	4 (26.7)	4 (26.7)	
Lenvatinib	11 (73.3)	11 (73.3)	
Etiology			0.700
Hepatitis B virus	10 (66.7)	8 (53.3)	
Hepatitis C virus	0 (0.0)	1 (6.7)	
Alcohol	3 (20.0)	3 (20.0)	
Autoimmune hepatitis	2 (13.3)	3 (20.0)	
Alpha-fetoprotein, ng/mL	15,270.8 ± 32,432.1	10,730.9 ± 19,279.8	0.645
Largest intrahepatic tumor size (cm)	9.2 ± 7.4	9.5 ± 5.4	0.908
Intrahepatic tumor number			0.111
Single	7 (46.7)	2 (13.3)	
Multiple	8 (53.3)	13 (86.7)	
Portal vein invasion	6 (40.0)	4 (26.7)	0.699
Extrahepatic metastasis	10 (66.7)	4 (26.7)	0.067
Creatinine (mg/dL)	1.1 ± 0.7	0.8 ± 0.3	0.094
Clinically significant portal hypertension	4 (26.7)	5 (33.3)	1.000
Child–Pugh score			0.705
5	9 (60.0)	8 (53.3)	
6	3 (20.0)	4 (26.7)	
7	3 (20.0)	2 (13.3)	
8	0 (0.0)	1 (6.7)	
Previous treatment history	14 (93.3)	9 (60.0)	0.084
Barcelona Clinic Liver Cancer stage			0.062
B	3 (20.0)	9 (60.0)	
C	12 (80.0)	6 (40.0)	
CD3 (Cell number/20,000 µm^2^)	77.1 ± 55.0	53.7 ± 43.4	0.207
CD68 (Cell number/20,000 µm^2^)	71.2 ± 42.3	39.3 ± 19.2	0.015
PD-L1 (Combined positive score)	9.1 ± 8.1	0.8 ± 0.4	0.001

Data are presented as *n* (%), mean ± SD, or median (quartiles). PD-L1, programmed cell death-ligand-1.

**Table 3:** Factors associated with overall survival analyzed by Cox regression analysis.

	Univariate Analysis		Multivariate Analysis	
Variable	HR (95% CI)	*p*-Value	HR (95% CI)	*p-*Value
CD3 (high vs. low)	0.794 (0.236–2.674)	0.710		
CD68 (high vs. low)	0.435 (0.118–1.611)	0.213		
PD-L1 (high vs. low)	0.731 (0.231–2.314)	0.594		
Child–Pugh score (>5)	4.167 (1.197–14.506)	0.025	4.269 (1.220–14.946)	0.023
Clinically significant portal hypertension	2.539 (0.731–8.812)	0.142		
Creatinine (>0.65 mg/mL)	0.825 (0.236–2.880)	0.763		
Alpha-fetoprotein (>200 ng/mL)	1.510 (0.484–4.709)	0.477		
Tumor size (>10 cm)	2.276 (0.676–7.661)	0.184		
Extrahepatic metastasis	0.504 (0.135–1.880)	0.307		

HR, hazard ratio; CI, confidence interval; PD-L1, programmed cell death-ligand-1.

**Table 4:** Factors associated with progression-free survival analyzed by Cox regression analysis.

	Univariate Analysis	
Variable	HR (95% CI)	*p*-Value
CD3 (high vs. low)	0.611 (0.227–1.642)	0.328
CD68 (high vs. low)	0.261 (0.084–0.813)	0.020
PD-L1 (high vs. low)	0.329 (0.119–0.907)	0.032
Child–Pugh score (>5)	3.071 (1.049–8.986)	0.041
Clinically significant portal hypertension	2.963 (0.962–9.129)	0.058
Creatinine (>0.65 mg/mL)	0.421 (0.079–2.232)	0.309
Alpha-fetoprotein (>200 ng/mL)	1.788 (0.703–4.543)	0.222
Tumor size (>10 cm)	0.905 (0.365–2.243)	0.830
Extrahepatic metastasis	0.807 (0.321–2.025)	0.648

HR, hazard ratio; CI, confidence interval; PD-L1, programmed cell death-ligand-1.

## Data Availability

The data presented in this study are available upon request from the corresponding author.
